# Nasopharyngeal Carcinoma and Its Effect on Dry Eye Disease: A Nationwide Cohort Study

**DOI:** 10.3390/ijerph20010387

**Published:** 2022-12-26

**Authors:** Ching-Tai Chen, Shun-Fa Yang, Shih-Chun Chao, Chia-Yi Lee, Jing-Yang Huang, Hung-Yu Lin

**Affiliations:** 1 Department of Ophthalmology, Show Chwan Memorial Hospital, Changhua 500, Taiwan; 2 Institute of Medicine, Chung Shan Medical University, Taichung 402, Taiwan; 3 Department of Medical Research, Chung Shan Medical University Hospital, Taichung 402, Taiwan; 4 Department of Optometry, Central Taiwan University of Science and Technology, Taichung 406, Taiwan; 5 Department of Optometry, Yuan Pei University, Hsinchu 300, Taiwan; 6 Department of Ophthalmology, Nobel Eye Institute, Taipei 115, Taiwan; 7 Department of Ophthalmology, Jen-Ai Hospital Dali Branch, Taichung 41265, Taiwan; 8 Department of Optometry, Chung Shan Medical University, Taichung 402, Taiwan; 9 Department of Post-Baccalaureate Medicine, College of Medicine, National Chung Hsing University, Taichung 402, Taiwan

**Keywords:** nasopharyngeal carcinoma, dry-eye disease, inflammation, risk factor, epidemiology

## Abstract

The aim of the current study was to investigate the relationship between nasopharyngeal carcinoma (NPC) and dry eye disease (DED) using the National Health Insurance Research Database (NHIRD) of Taiwan. A retrospective cohort study was conducted, and patients with an NPC diagnosis were included. Next, one NPC patient was matched to four non-NPC participants via demographic data and systemic comorbidities. In total, 4184 and 16,736 participants were enrolled in the NPC and non-NPC groups, respectively. The primary outcome was the development of DED one year after the diagnosis of NPC. Cox proportional hazard regression was applied to estimate the adjusted hazard ratios (aHRs) with 95% confidence intervals (CIs) of DED. In this study, 717 and 2225 DED cases were found in the NPC and non-NPC groups, respectively, and the NPC group showed a significantly higher incidence of DED development compared to the non-NPC group (aHR: 1.45, 95% CI: 1.33–1.58, *p* < 0.0001) in the multivariable analysis. The other covariates that were positively correlated with DED development included age over 40 years, an education level higher than senior high school, hypertension, DM, allergic pulmonary diseases, allergic otolaryngologic diseases, and allergic dermatological diseases (all *p* < 0.05). In conclusion, the presence of NPC is an independent risk factor for subsequent DED.

## 1. Introduction

Nasopharyngeal carcinoma (NPC) is an epithelial cancer arising from the nasopharyngeal region [1]. It is endemic in East and Southeast Asia [2]; while the prevalence of NPC is about 0.4 per 100,000 persons in Western countries, it rises to 3.0 per 100,000 individuals in the Chinese population [3]. The Epstein–Barr virus, human papillomavirus, and tobacco smoking were shown to be causally linked to the development of NPC [1,3,4]. Currently, the main treatments for NPC are radiotherapy and chemotherapy, with a recurrence rate of approximately 10–20 percent [5,6], and the median of survival period is 3 years in the advanced stage, which could be influenced by marital status [7,8].

The presence of NPC and its therapy could cause local invasion and damage adjacent tissue [9]. Cervical masses due to lymphadenopathy are common findings in NPC [9], and chronic rhinosinusitis was found to correlate with NPC development [10]. In addition, radiotherapy in NPC and NPC itself can contribute to injury to the auditory system, including otitis media with effusion and sensorineural hearing loss [9,11]. On the other hand, NPC is also associated with swallowing difficulty after radiotherapy and central-nervous-system disorders, such as skull-base osteoradionecrosis, cavernous sinus thrombosis, and meningitis, have been reported after the diagnosis of NPC [12,13].

Due to its close location, the eye is a common site of NPC-related complications [14]. In previous research, orbital involvement was found in 1.1 percent of patients with NPC, accounting for about half of all the ophthalmic involvements of NPC [15]. Furthermore, the radiotherapy used in NPC treatment is related to cataract and radiation retinopathy [16]. Dry-eye disease (DED) is a common inflammatory ocular disease that features unstable tear film, dryness, and ocular irritation [17,18]. Nevertheless, the possible relationship between NPC and DED has not been reported elsewhere with adequate patient numbers. Both NPC and DED cause inflammatory responses and NPC-related damage to the lacrimal gland has been described [3,18,19]. Ophthalmic involvement during NPC progression can change the ocular structure and lead to proptosis, which may enhance the risk of DED [14]. Moreover, alacrima, which means loss of tears, was reported as an early sign in a patient subsequently diagnosed with NPC [20]. Nasopharyngeal carcinoma and DED have a high prevalence worldwide, especially in the Asian population [1,18]; thus, patients with NPC may benefit from additional DED exams if DED is indeed more prevalent in NPC. Consequently, the potential relationship between NPC and DED should be investigated with adequate case numbers.

To support our notion that the presence of NPC may influence the incidence of DED, we aimed to survey the possible relationship between NPC and DED using the National Health Insurance Research Database (NHIRD) of Taiwan.

## 2. Materials and Methods

### 2.1. Data Source

We conducted a retrospective cohort study, and all the procedures and methods in this study adhered to the declaration of Helsinki in 1964 and later amendments. Our study was approved by both Institutional Review Board of Chung Shan Medical University Hospital (project identification code: CS1-20108), and National Health Insurance Administration, and the necessity of signed informed consent was waived by the aforementioned institutions. The NHIRD in Taiwan owns claimed data of health insurance for roughly the whole Taiwanese population-approximately 23 million participants. The research period of NHIRD in this version ranged from the 1 January 2000 to the 31 December 2016. The International Classification of Diseases, Ninth Revision (ICD-9) diagnostic code, medical-department type, age, sex, education level, location, image, laboratory examination code, procedure and surgery codes, and international ATC codes for medications can be obtained from the NHIRD. In this study, we applied the longitudinal health-insurance database (LHID) 2000 version, which is a sub-database of NHIRD, for all the analyses. In the LHID 2000, roughly two million patients were randomly sampled from the NHIRD in the year of 2000, and all the data available in NHIRD were also be obtained in the LHID 2000. Furthermore, the patients in the LHID 2000 were followed over the same time period as in the NHIRD, from 1 January 2000 to 31 December 2016.

### 2.2. Patient Selection

The participants were regarded as NPC if they fulfilled the following criteria: (1) receipt of NPC-related ICD-9 diagnostic codes (147.x), (2) the arrangement of sinoscopy and biopsy before the diagnosis of NPC, (3) the arrangement of Epstein–Barr virus DNA exam before the diagnosis of NPC, (4) a follow-up of at least 3 years, and (5) NPC diagnosis by an otorhinolaryngologist. The time of index date was the date one year after the NPC diagnosis. To make the general condition of our study population more homogenous, the following exclusion criteria were obeyed to discard patients with extreme morbidity: (1) age younger than 20 years old, (2) blindness before the index date, (3) the diagnosis of ocular tumor before index date, (4) the receipt of eyeball removal before the index date, (5) the receipt of corneal transplantation before the index date, (6) achievement of outcome before index date, (7) died before index date. After the exclusion process, we performed two matching processes for the constitution of NPC and non-NPC groups. In the first step, we matched the remaining NPC patients to non-NPC patients in the LHID 2000 via age and sex at a ratio of 1:8, and 5687 and 45,496 patients constituted the first NPC and non-NPC groups, respectively. Next, one patient in the first NPC group (i.e., the 5687 NPC patients) was matched to four non-NPC patients in the first non-NPC groups (i.e., the 45,496 non-NPC individuals) by the propensity score matching (PSM) method. The covariates included in the PSM included demographic data (including sex, age, marital status, and education level) and systemic comorbidities (including hypertension, diabetes mellitus, coronary artery disease, hyperlipidemia, cerebrovascular disease, allergic pulmonary diseases, rheumatic disease, allergic otolaryngologic diseases, and allergic dermatological diseases). The NPC patient in the first NPC group that could not be matched to four non-NPC patients in the first non-NPC group was excluded. Finally, 4184 and 16,736 patients constituted the final NPC and non-NPC groups after PSM.

### 2.3. Primary-Outcome Measurement

The primary outcome in this study was regarded as the development of DED, for which the following criteria were set: (1) the receipt of DED-related ICD-9 diagnostic codes (370.33, 370.34, 372.53, 375.15, 710.2), (2) the arrangement of fluorescein staining of cornea or Schirmer test before or on the day of DED diagnosis, (3) the prescription of artificial tear after the DED diagnosis, and (4) DED diagnosis by an ophthalmologist. To clarify the temporal relationship between NPC and DED, only those diagnosed with DED one year after the index date were regarded as the occurrence of primary outcome. All study individuals were at risk at index date and followed from the index date until development of DED or conclusion of treatment.

### 2.4. Demographic and Comorbidity Covariates

To evaluate and adjust for potential confounders of DED, the following confounders were included in the multivariable model: age (ID_BIRTH_Y), sex (ID_S), marriage status (ID1_MARRIAGE), insurance fee (ID1_AMT), hypertension (ICD-9 codes: 401.x-405.x), diabetes mellitus (DM) (ICD-9 codes: 250.x, 277.7), stable coronary arterial disease (CAD) (ICD-9 codes: 410.x, 412.x, 414.0, 414.0.x, 414.2, 414.3, 414.4, 414.8, 414.9), hyperlipidemia (ICD-9 codes: 272.0, 272.1, 272.2, 272.4, 272.9), cerebrovascular disease (ICD-9 codes: 430.x–438.x), allergic pulmonary diseases (ICD-9 codes: 490, 491.x, 493.x, 495.x, 500-505), rheumatic disease (ICD-9 codes: 446.5, 710.0, 710.1, 710.3, 710.4, 714.0–714.2, 714.8, 725.x), allergic otolaryngologic diseases (ICD-9 codes: 473.0-473.3, 477.x), and allergic dermatological diseases (ICD-9 codes: 691.8, 692.0-692.6, 692.8, 692.9, 693.x, 708.x). The age was continuous covariate, while sex, marital status and insurance fee were categorical covariates, and systemic diseases were binary covariates. In addition, only those systemic diseases persisted more than two years were included in the multivariable analyses. We followed our patients until outcome achievement, they left the National Health Insurance Program, or the end of LHID 2000, which means 31 December 2016.

### 2.5. Statistical Analysis

In our study, we applied SAS version 9.4 (SAS Institute Inc, Cary, NC, USA) for all the statistical analyses. The sample-size calculation revealed a high statistical power of 1.00, which was estimated according to the study numbers and risk ratio. Descriptive analyses with absolute standardized difference (ASD) were used to show the distribution of basic characteristics across the NPC and non-NPC groups. An ASD value of more than 0.1 was defined as statistically significant in our study. The number of main treatments for NPC patients, which included radiotherapy and chemotherapy, was also shown via descriptive analyses. Next, we applied Poisson regression for the incidence ratio of DED and conducted Cox proportional hazard regression for the adjusted hazard ratios (aHR) and corresponding 95% confidence intervals (CI) of DED between the two groups. We tested the assumption of proportional hazard for the risk of DED, and the assumption was not rejected. The crude hazard ratio and aHR, including the co-variates of demographic data and systemic comorbidities, were estimated by univariate and multivariable Cox regression analysis, respectively. We did not apply stratified analysis. Furthermore, the effect of each covariate was calculated by the Cox proportional hazard regression again. The Kaplan–Meier curve was painted to show the cumulative probability of DED between the two groups, and we used the log-rank test for surveying the significance between the two Kaplan–Meier curves. In addition, the incidences of DED among NPC patients with different doses of radiotherapy, NPC patients without radiotherapy, and the control groups were compared via Cox proportional hazard regression. A similar comparison was conducted between NPC patients with chemotherapy, NPC patients without chemotherapy, and control group. The statistical significance was determined as *p* < 0.05 in our study, and a *p* value of less than 0.0001 was delineated as *p* < 0.0001.

## 3. Results

### 3.1. Clinical Characteristics in the Non-NPC and NPC Groups

**Figure 1:**
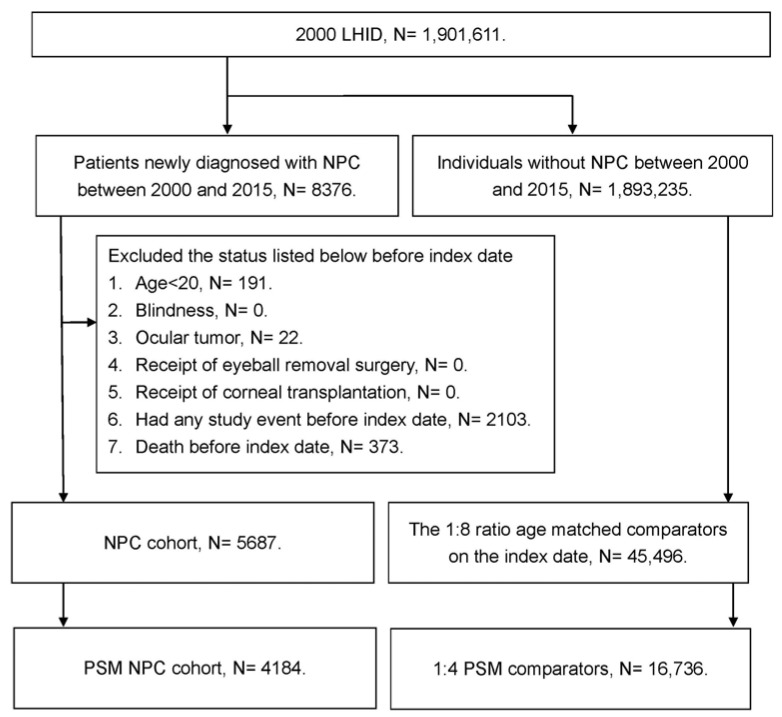
The flow chart of the patient selection. NPC: nasal pharyngeal carcinoma, N: number, PSM: propensity-score matching.

The flow chart for the patient selection is shown in Figure 1. In total, 4184 NPC patients and 16,736 non-NPC patients were enrolled in the NPC group and non-NPC group, respectively. The basic characteristics of the NPC and non-NPC groups are shown in Table 1. The mean and standard deviation (SD) of age in the NPC group was 49.7 ± 14.1, which was similar to that in the non-NPC group (mean ± SD: 49.0 ± 13.8, ASD: 0.0567). There were 63.26 percent male patients in the NPC group, which was also similar to the 64.44 percent male patients in the non-NPC group (ASD: 0.0244). The other parameters, including marital status, education level, and the distribution of comorbidities, also showed insignificant differences between the NPC and non-NPC groups due to the PSM procedure (all ASD < 0.1) (Table 1). In total, 1584, 922, and 934 NPC patients received chemotherapy, low-dose radiotherapy, and high-dose radiotherapy, respectively (Table 1).

### 3.2. Risk of DED in NPC and Non-NPC Groups

**Figure 2:**
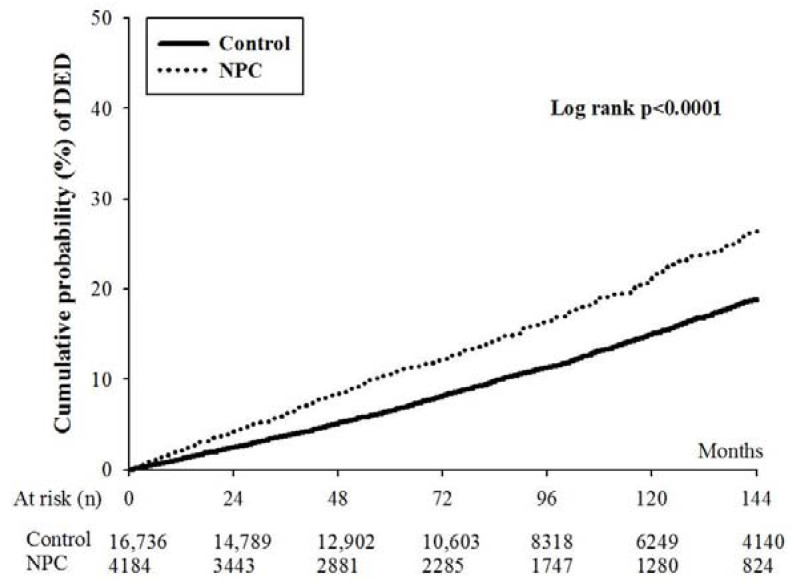
The Kaplan–Meier curves of the nasal pharyngeal carcinoma and non-nasal pharyngeal carcinoma populations.

There were 717 (17.14%) and 2225 (13.29%) episodes of DED in the NPC and non-NPC groups after the whole study interval (Table 2). Even after the adjustment of potential confounders via Cox proportional hazard regression, the patients with NPC still demonstrated a significantly higher incidence of DED development compared to the non-NPC individuals (aHR: 1.45, 95% CI: 1.33–1.58, *p* < 0.0001). The Kaplan–Meier curve revealed a significantly higher cumulative probability of DED in the NPC group compared to the non-NPC group (*p* < 0.0001) (Figure 2). The other covariates that significantly correlated with DED development (all *p* < 0.05) were listed in Table 3.

### 3.3. The Effect of Radiotherapy and Chemotherapy on the Development of DED

Regarding the effect of radiotherapy on the development of DED, the aHR among NPC patients without radiotherapy, NPC patients with low-dose radiotherapy, and NPC patients with high-dose radiotherapy were similar (95% CIs of the latter two included 1). However, the non-NPC patients revealed a significantly lower ratio of DED compared to the NPC patients without radiotherapy (aHR: 0.705, 95% CI: 0.645–0.770; Table 4). On the other hand, the NPC patients with chemotherapy showed a higher aHR for DED than the NPC patients without chemotherapy (aHR: 1.456, 95% CI: 1.098–1.931), while the aHR for the DED in the non-NPC population was significantly lower than that in the NPC population without chemotherapy (aHR: 0.693, 95% CI: 0.636–0.756) (Table 5).

## 4. Discussion

Briefly, the current study showed a significant correlation between NPC and subsequent DED. The other factors that significantly influenced DED development included old age, male sex, high education level, widowed condition, hypertension, DM and several allergic diseases.

Nasopharyngeal carcinoma affects the tissue around the skull base and the nasal-pharyngeal regions, as well as causing significant damage to both orbital and ocular tissues [13,15,19]. Nasopharyngeal carcinoma can invade the orbit via the pterygopalatine fossa, the inferior orbital fissure, and the orbital apex region, and contributes to subsequent diplopia, eyelid swelling, and extraocular muscle limitation [19]. Furthermore, NPC can change the position of ocular tissue position and subsequently cause proptosis and cranial-nerve palsy [15]. In certain cases, the growth of NPC-related tissue in the orbital region can lead to a compressive effect and associated optic neuropathy [15]. In such circumstances, visual acuity is severely and irreversibly impaired [21]. Regarding the relationship between NPC and inflammation, interleukin polymorphism was proposed as a risk factor for NPC [22], and inflammation in the sinonasal tract is correlated with subsequent NPC [23].

On the other hand, DED had been proposed as a target of treatment in recent years [24]. The systemic diseases that correlate with the development of DED include rheumatic arthritis, systemic lupus erythematous, Sjogren syndrome, and systemic sclerosis [17,25,26]. Recently, gout was found to elevate the risk of DED development, with longer periods of gout causing higher rates of DED [27]. In terms of local inflammatory disease, the presence of chronic rhinosinusitis is correlated with subsequent DED occurrence after excluding the effect of multiple risk factors for DED [28]. This is because the orbital and ocular involvement in NPC progression can alter the ocular structure and cause proptosis in the majority of cases [14], and NPC itself or radiotherapy can lead to ocular damage and possible inflammation [3,29]. We speculated that although NPC cannot directly lead to the development of DED, NPC may elevate the risk of DED due to structural and inflammatory elements. This hypothesis was partially supported by the results of the current study.

In this study, the presence of NPC was associated with subsequent DED development. To our knowledge, this is a relatively novel finding, which further demonstrates the wide influence of NPC on the ocular region. Moreover, we adjusted several risk factors for DED, including age, sex, marital status, DM, and certain allergic diseases, in the statistical analyses in our study. The results illustrated that NPC is an independent risk factor for DED after erasing the effect of other prominent confounders. Furthermore, only the occurrence of DED one year after the diagnosis of NPC was regarded as an outcome. Moreover, the cumulative probability obtained from the Kaplan–Meier curve showed a progressively higher DED incidence as the disease interval of NPC increased compared to the non-NPC population. Concerning the effect of radiotherapy and chemotherapy on the development of DED in NPC populations, the administration of radiotherapy did not alter the incidence of DED. Although the NPC patients with chemotherapy were found to be at a higher risk of DED than the NPC patients without chemotherapy, the incidence of DED in the NPC patients without chemotherapy was still higher than in the non-NPC population, with statistical significance. Consequently, our results further indicated the significant effect of NPC on the occurrence of DED, suggesting that patients with prolonged NPC are at higher risk. In previous studies, DED was correlated with several inflammatory diseases [17,18,30], while a relationship between DED and malignancy was seldom presented. We proposed that although the malignancy did not cause significant systemic inflammation compared to systemic inflammatory diseases such as rheumatic arthritis or systemic lupus erythematous [17], the immune and inflammatory response of NPC itself could contribute to the elevation of inflammation in nearby regions, as described in previous studies [22,31]. Since both NPC and DED feature a high prevalence in the Asian population [1,18], this relationship should be illustrated. Nevertheless, further study is needed to clarify the potential relationship we propose.

Regarding the other risk factors for DED in this study, old age is a known risk factor [18,30]. In previous studies, the incidence of DED was about 11 percent in 50-year-old populations, rising to around 15 percent in individuals aged 85 years or more [24,32]. A higher educational level, which refers to senior high school or above, was associated with DED in our study. We speculated that patients with higher educational levels were more likely toto work with visual display monitors, which is a significant predisposing factor for DED [30]. Regarding systemic diseases, hypertension and DM are metabolic disorders that can trigger DED [33]. The relationship between ocular allergic diseases and DED may be due to the surge of inflammatory cytokines and damage to the ocular surface [34]. This may explain the significant effect of these allergic diseases on DED in our study.

There are some limitations in our study. Firstly, the database-processing nature of our research limited the accessibility of several important data, including the stage of NPC, the biopsy results for NPC, the radiographic findings of NPC, the treatment effect and postoperative image results for NPC, the recurrence of NPC, whether the NPC tissue involved orbital and ocular areas, the severity of DED, the results of DED-related examinations, imaging, including external-eye photography, of those with DED, and the exact therapeutic program/agents for DED, since we could only track the patients until the day of their DED diagnosis. Furthermore, because nearly all the NPC participants in our research received radiotherapy, we could not decide whether the DED resulted from the NPC itself or the radiotherapy-related injury to the orbital area and lacrimal gland. In addition, the retrospective design of our study diminished the homogeneity of our study, despite the fact that we used PSM to compensate. Furthermore, the most recent data (i.e., from late 2010 to 2022) have not been released by the National Health Insurance Administration, which may have influenced the results of our analyses. In addition, more than half of the NPC patients were dismissed during our exclusion-and-matching process, which reduced the statistical power. However, the participant number in the NPC group in our study was above 4000, which is not inferior to those in previous studies [15,19]. Consequently, the influence of statistical power might not be significant.

## 5. Conclusions

In conclusion, the presence of NPC may be correlated with subsequent DED. Furthermore, this correlation is significant after erasing the influence of several risk factors. Consequently, patients with persistent NPC may be at risk of this condition and referred to the ophthalmic department if related symptoms develop. Further large-scale prospective studies to evaluate the presence of NPC and to monitor DED severity are mandatory.

## Figures and Tables

**Table 1:** Characteristics of nasal pharyngeal carcinoma patients and control group after propensity-score matching.

Characters	Non-NPC Group	NPC Group	ASD
N	16,736	4184	
Sex			0.0244
Female	5952 (35.56%)	1537 (36.74%)	
Male	10784 (64.44%)	2647 (63.26%)	
Age (mean ± SD)	49.0 ± 13.8	49.7 ± 14.1	0.0567
20–30	1349 (8.06%)	326 (7.79%)	
30–40	2914 (17.41%)	687 (16.42%)	
40–50	4485 (26.80%)	1100 (26.29%)	
50–60	4363 (26.07%)	1078 (25.76%)	
60–70	2285 (13.65%)	607 (14.51%)	
70–80	1032 (6.17%)	300 (7.17%)	
80–100	308 (1.84%)	86 (2.06%)	
Marital status			0.0532
Unmarried	2868 (17.14%)	684 (16.35%)	
Married	12,262 (73.27%)	3063 (73.21%)	
Divorced	995 (5.95%)	262 (6.26%)	
Widowed	611 (3.65%)	175 (4.18%)	
Education level			0.0447
Elementary school or below	4851 (28.99%)	1264 (30.21%)	
Junior high school	3023 (18.06%)	775 (18.52%)	
Senior high school	6916 (41.32%)	1666 (39.82%)	
University or above	1946 (11.63%)	479 (11.45%)	
Comorbidities			
Hypertension	3148 (18.81%)	851 (20.34%)	0.0386
Diabetes mellitus	1567 (9.36%)	407 (9.73%)	0.0124
Stable CAD	683 (4.08%)	210 (5.02%)	0.0450
Hyperlipidemia	1529 (9.14%)	424 (10.13%)	0.0338
Cerebrovascular disease	752 (4.49%)	201 (4.80%)	0.0148
Allergic pulmonary diseases	1348 (8.05%)	456 (10.90%)	0.0972
Rheumatic disease	132 (0.79%)	46 (1.10%)	0.0321
Allergic otolaryngologic diseases	398 (2.38%)	99 (2.37%)	0.0008
Allergic dermatological diseases	3240 (19.36%)	843 (20.15%)	0.0198
Chemotherapy		1584 (37.9%)	
Low dose radiotherapy		922 (22.0%)	
High dose radiotherapy		934 (22.3%)	

NPC: nasal pharyngeal carcinoma, N: number, ASD: absolute standardized difference, SD: standard deviation, DM: diabetes mellitus, CAD: coronary arterial disease.

**Table 2:** Study event between the nasal pharyngeal carcinoma and non-nasal pharyngeal carcinoma patients.

Outcome	Non-NPC Group	NPC Group
N	16736	4184
Person-months	1603235	353946
Event	2225	717
Crude HR (95% CI) *	Reference	1.48 (1.36–1.61)
aHR (95% CI)	Reference	1.45 (1.33–1.58)

N: number, NPC: nasal pharyngeal carcinoma, CI: confidence interval, aHR: adjusted hazard ratio which including demographic and systemic comorbidity variables. * Crude incidence rate, per 10,000 person-months.

**Table 3:** Cox regression for estimating the hazard ratio of dry-eye diseases.

Parameters	aHR (95% CI)	*p* Value
NPC	1.45 (1.33–1.58)	<0.0001 *
Sex (ref = Female)		
Male	0.45 (0.42–0.49)	<0.0001 *
Age (ref = 30–40)		
20–30	0.88 (0.72–1.08)	0.2175
40–50	1.32 (1.16–1.50)	<0.0001 *
50–60	1.98 (1.74–2.26)	<0.0001 *
60–70	2.31 (1.98–2.71)	<0.0001 *
70–80	2.36 (1.94–2.88)	<0.0001 *
80–100	1.73 (1.20–2.50)	0.0034*
Marital status (ref = Married)		
Unmarried	0.92 (0.80–1.07)	0.2730
Divorced	0.93 (0.79–1.10)	0.4004
Widowed	0.83 (0.69–0.98)	0.0295 *
Education (ref = Junior high school)		
Elementary school or below	1.02 (0.90–1.15)	0.7483
Senior high school	1.18 (1.06–1.33)	0.0040 *
University or above	1.44 (1.24–1.67)	<0.0001 *
Comorbidities		
Hypertension	1.14 (1.03–1.26)	0.0130 *
DM	1.17 (1.03–1.32)	0.0140 *
Stable CAD	1.08 (0.92–1.28)	0.3360
Hyperlipidemia	1.09 (0.96–1.24)	0.1651
Cerebrovascular disease	1.02 (0.86–1.21)	0.8558
Allergic pulmonary diseases	1.18 (1.05–1.32)	0.0060 *
Rheumatic disease	1.20 (0.88–1.63)	0.2547
Allergic otolaryngologic diseases	1.28 (1.05–1.55)	0.0136 *
Allergic dermatological diseases	1.22 (1.12–1.33)	<0.0001 *

aHR: adjusted hazard ratio which including demographic and systemic comorbidity variables, NPC: nasal pharyngeal carcinoma, CI: confidence interval, DM: diabetes mellitus, CAD: coronary arterial disease. * Denotes significant correlation between parameter and dry-eye disease.

**Table 4:** Outcomes for the patients with nasal pharyngeal carcinoma stratified by radiotherapy and individuals without nasal pharyngeal carcinoma.

Outcome	Non-NPC Group (N = 16,736)	NPC Without Radiotherapy (N = 2328)	NPC With Low-Dose Radiotherapy (N = 922)	NPC With High-Dose Radiotherapy (N = 934)
Person-months ^#^	1,603,235	322,389	17,834	13,723
DED event	2225	635	52	30
aHR (95% CI)	0.705 (0.645–0.770) *	Reference	1.528 (0.733–2.323)	1.110 (0.770–1.601)

DED: dry-eye disease, NPC: nasal pharyngeal carcinoma, N: number, CI: confidence interval, aHR: adjusted hazard ratio which including demographic and systemic comorbidity variables. ^#^ Crude incidence rate, per 1000 person-months. * Denotes significant difference compared with reference group.

**Table 5:** Outcomes for the patients with nasal pharyngeal carcinoma stratified by chemotherapy and individuals without nasal pharyngeal carcinoma.

Outcome	Non-NPC Group (N = 16,736)	NPC Without Chemotherapy (N = 2600)	NPC With Chemotherapy (N = 1584)
Person-months ^#^	1,603,235	330,107	23,839
Event of DED	2225	661	56
aHR (95% CI)	0.693 (0.636–0.756) *	Reference	1.456 (1.098–1.931) *

NPC: nasal pharyngeal carcinoma, N: number, CI: confidence interval, aHR: adjusted hazard ratio which including demographic and systemic comorbidity variables. ^#^ Crude incidence rate, per 1000 person-months. * Denotes significant difference compared with reference group.

## Data Availability

Restrictions apply to the availability of these data. Data were obtained from the National Health Insurance database and are available from the authors with the permission of the National Health Insurance Administration of Taiwan.
